# Effect of transcutaneous electrical acupoint stimulation on protecting against radiotherapy- induced ovarian damage in mice

**DOI:** 10.1186/s13048-019-0541-1

**Published:** 2019-07-19

**Authors:** Rongrong Tan, Yuheng He, Suyun Zhang, Danhua Pu, Jie Wu

**Affiliations:** State Key Laboratory of Reproductive Medicine, Department of Obstetrics and Gynecology, The First Affiliated Hospital of Nanjing Medical University, Nanjing Medical University, Nanjing, 210029 People’s Republic of China

**Keywords:** Irradiation protection, Transcutaneous electrical acupoint stimulation (TEAS), Ovarian damage

## Abstract

**Background:**

Premature ovarian insufficiency (POI) is characterized by early loss of ovarian function that affects women before the age of 40. We aim to explore the protective effects of transcutaneous electrical acupoint stimulation (TEAS) against irradiation-induced ovarian damage in mice.

**Methods:**

C57BL6 mice were randomly divided into control and irradiation (IR) groups. Then, control group was divided into two treatment subgroups: mock TEAS treatment (control-) and TEAS treatment (control+). IR group was divided into four subgroups according to the time of treatment started: mock TEAS treatment initiated at 2 days after irradiation (IR 2D-), TEAS treatment initiated at 2 days after irradiation (IR 2D+), mock TEAS treatment initiated at 1 week after irradiation (IR 1 W-), and TEAS treatment initiated at 1 week after irradiation (IR 1 W+). The radiation model mice were exposed to single whole body X-ray irradiation (4 Gy), and the control mice received 0 Gy. TEAS stimulation (2 Hz, 1 mA, 30 min/day) was given once a day for six consecutive days per week for 2 weeks. Estrous cycle, ovarian weight, serum AMH level and follicle counts were evaluated. Then, proliferation markers, apoptotic markers and oxidative stress markers were examined.

**Results:**

Compared with the control group, the estrous cycle was disordered, and the ovarian weight, serum AMH, and primordial, primary and secondary follicles counts decreased (all *P* < 0.01) in the IR 2D- and IR 1 W- groups. In the irradiation with early TEAS treatment group (IR 2D+), the estrous cycle improved, the AMH level and primordial follicular significantly increased compared to the irradiation with mock group (IR 2D-). However, there were no significant differences in the estrous cycle, AMH level and follicle counts between IR 1 W- and IR 1 W+ groups. Moreover, IR 2D+ mice reduced the expression of Bax protein and increased the levels of Bcl-2 and PCNA compared to the IR 2D- group. Furthermore, the early TEAS treated mice showed significantly lower levels of oxidative stress and number of TUNEL (+) granulosa cells than that in the IR 2D- group.

**Conclusion:**

This study is first to evaluate TEAS as a potential therapy to attenuate irradiation-induced ovarian failure through inhibiting primordial follicles loss, increasing serum AMH secretion, inducing antioxidant, and anti-apoptotic systems.

## Background

Premature ovarian insufficiency (POI) is a hypergonadotropic ovarian deficiency with elevated gonadotropins and low estrogen levels that affects approximately 1% women before the age of 40 [1]. The underlying etiology for POI is complex and remains to be revealed. Our previous studies have found that HFM1 gene mutations and ESR1 gene polymorphisms may be the predisposing factors of idiopathic POI [2, 3]. Radiation therapy is one of the important etiologies for POI, which has well-documented toxic effects on the reproductive system and the fertility of women [4]. In addition, the ovary is highly sensitive to radiation, and primordial follicles appear to be the most sensitive among all stages of follicles [5]. Generally, the major mechanisms of ovarian injuries include follicle cell apoptosis, oxidative stress, ovarian atrophy, cortical fibrosis and blood-vessel damage [6–8]. However, radiation causes damage mostly via ionization and formation of reactive oxygen species (ROS), and the increased ROS induces rapid primordial follicle loss in the ovaries [9, 10]. The hallmarks of radiation-induced POI are menstrual disorder, infertility, osteoporosis, psychological problems, and impaired quality of life [1]. POI is irreversible, but early detection of ovarian failure allowing for a timely diagnosis in conjunction with early treatment can possibly delay or even improve the condition. Therefore, finding an effective and safe protective strategy is critical for both young cancer patients and reproductive scientists.

Recently, many studies in the field of reproductive medicine showed that transcutaneous electrical acupoint stimulation (TEAS) might provide an important methodology for restoring ovarian function [11–14]. TEAS is a noninvasive treatment combining acupuncture point stimulation with electrical stimulation, which is easily acceptable for patients who are afraid of the pain and topical skin tissue injury caused by acupuncture.

Mounting evidence has shown that follicular atresia is generally due to the apoptosis of granulosa cells [15]. Radiation not only causes DNA damage but also leads to apoptosis through mitochondrion-dependent apoptotic signaling in ovarian follicles [16, 17]. At the mitochondrial level, Bcl-2 family proteins play a central role in the regulation of apoptosis. Among them, Bax, a pro-apoptotic factor, and Bcl-2, an anti-apoptotic factor, are important in deciding the fate of cells [18]. In addition, proliferating cell nuclear antigen (PCNA) has been suggested to be a key regulator during the development of ovarian follicles, and it is known as the proliferation marker in follicles [19]. The PCNA-positive granulosa cells were decreased in association with follicular atresia after exposure to radiation in mice [20]. Furthermore, radiation can induce obvious ovarian toxicity with TUNEL (terminal deoxynucleotidyl transferase dUTP nick end labeling)-positive cells seen in secondary, preantral, and antral follicles in vivo [20]. Therefore, it has been reported that radiation impairs the ovaries by triggering apoptosis of follicular cells and inhibiting proliferation [20, 21].

The protective effect of TEAS on radiation-induced damage in organs (such as brain and salivary glands) has been studied in recent years [22, 23]. However, no such studies have been performed on the protective effect of TEAS on ovarian function in mice exposed to whole body radiation. Therefore, our study aimed to investigate the ovarian protective effects of TEAS in mice exposed to total irradiation.

## Methods

### Animals and experimental design

Eight-week-old female C57BL/6 J mice (*n* = 60), weighing 18.0 to 20.0 g, were purchased from the Animal Core Facility of Nanjing Medical University (Nanjing, China). They were housed in a temperature- and humidity-controlled room and maintained with a 12-h light/dark cycle. All animals were acclimatized for 1 week before the experiment. The mice were kept at a constant temperature of 23 °C ± 2 °C and humidity of 55% ± 5%, and they were fed with standard pelleted food and tap water.

**Fig. 1:**
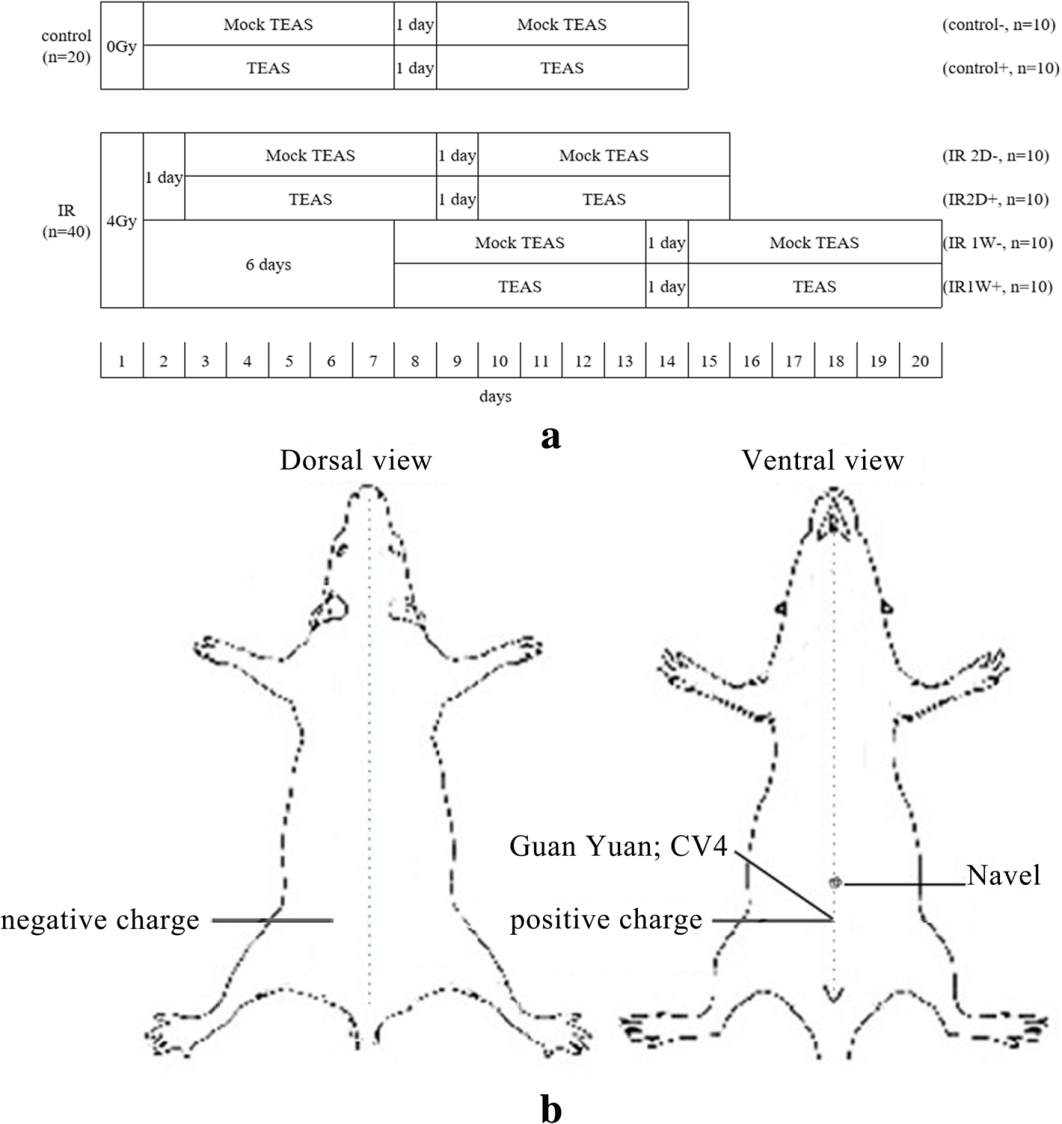
**a** Schematic diagram of the experimental design. **b** Schematic drawing showing the location of Guan Yuan (CV4) and electrode sheets

As shown in Fig. 1a, sixty mice were randomly divided into control (*n* = 20) and IR (*n* = 40) groups. Then, control group was divided into two treatment subgroups: mock TEAS treatment (control-, *n* = 10) and TEAS treatment (control+, *n* = 10). IR group was divided into four subgroups (*n* = 10 for each group) according to the time of treatment started: mock TEAS treatment initiated at 2 days after irradiation (IR 2D-), TEAS treatment initiated at 2 days after irradiation (IR 2D+), mock TEAS treatment initiated at 1 week after irradiation (IR 1 W-), and TEAS treatment initiated at 1 week after irradiation (IR 1 W+).

### X-ray irradiation

The X-ray irradiation was performed using a biological X-ray irradiation apparatus (R 000Pro; Rad source, USA) at Animal Core Facility of Nanjing Medical University. The model mice received whole-body X-ray irradiation at a single dose of 4 Gy (the intensity of 1.2 Gy/min), and the control mice received 0 Gy based on the study of Gao et al. [24].

### Treatment with TEAS

The mice were anesthetized with an intraperitoneal injection of 4% chloral hydrate (0.4 g/kg) to minimize any restraint stress before TEAS treatment. The mice received TEAS through self-adhesive surface electrodes using a TEAS treatment device (RYZUR-NT6021; Nanjing Jisheng Medical Technology Co., China). After removing the hair and wiping the skin with ethyl alcohol, the positive charge (5 mm diameter) was attached to the acupoint of Guanyuan (CV 4, located on the midline of the lower abdomen, 10 mm inferior to the umbilicus) [25, 26], and the negative charge was connected to skin of lateral spine (Fig. 1b). Electrical stimulation lasted for 30 min using a continuous wave at a low frequency of 2 Hz (200 μs pulse width) and a current of 1 mA. These parameters of TEAS treatment were in accordance with those used in clinical practice. The TEAS treatment was administered once a day for six consecutive days per week for 2 weeks. The three mock TEAS treatment subgroups were given all manipulations without electrical stimulation.

### Vaginal cytology

For analysis of the estrus cycle, vaginal smears were obtained every day between 08:30 and 09:00 during the TEAS treatment period. After the samples were stained with toluidine blue, they were analyzed under a light microscope (Axio ImagerA1, Carl Zeiss, Germany) by two independent researchers. If the researchers disagreed, a final result was reached by discussion. Cytological appearance of estrous phase was determined as follows: 1) The proestrous phase consists of predominance of nucleated epithelial cells. 2) An estrous phase primarily consists of anucleated cornified cells. 3) An metestrous phase indicated by equal proportions of leucocytes, anucleated cornified and nucleated epithelial cells. 4) The diestrous phase primarily consists of predominance of leucocytes according to previous description [27].

### Tissue collection and processing

On the last day of the TEAS treatment, 4% chloral hydrate (0.4 g/kg) was intraperitoneally injected into all mice. Blood was collected and separated by centrifugation at 3 000 g for 15 min; then, serum was stored at − 80 °C until assessment of AMH. All ovarian samples were free of oviduct, fat, and bursa. After they were weighed, the ovaries from one side were immediately stored at − 80 °C until protein quantification and oxidative stress markers analysis; the ovaries from the other side were fixed in 4% paraformaldehyde for hematoxylin and eosin (H&E) staining and TUNEL immunostaining.

### Measurement of serum AMH

Serum AMH concentration was performed with mouse ELISA kit for AMH (CUSABIO, CSB-E13156m; USA). The standards were performed in duplicate, and the samples were performed in triplicate. The assays were performed according to the manufacturer’s protocol, and the concentration of AMH was determined from the standard curve.

### Ovarian histology and follicle counting

Ovaries (1 per mouse) fixed in 4% paraformaldehyde were embedded in paraffin wax and were serially sectioned at 5 μm thickness throughout the entire ovary. After H&E staining, blind follicle counts were conducted on every fifth section of the entire ovary by two independent researchers and were scored as primordial, primary, secondary, or antral follicles, based on their morphological appearance, as detailed in the literature [28, 29]: (a) primordial follicle contains an oocyte surrounded by a partial or complete layer of squamous granulosa cells, (b) primary follicle comprises an oocyte surrounded by a single layer of cuboidal granulosa cells, (c) secondary follicle consists of an oocyte surrounded by more than one layer of cuboidal granulosa cells and no visible antrum, and (d) antral follicle has a clearly defined antral space and cumulus granulosa cell layer. The total follicle count per ovary was calculated. Evaluating radiation-dependent cellular damage, vascularity and fibrosis were used to determine the effects of irradiation and TEAS treatment on the ovary. Atretic follicles were identified due to the presence of a degenerating oocyte or pyknotic granulosa cells.

### Protein quantification by Western blotting

Mouse ovaries were homogenized ultrasonically in radio immunoprecipitation assay (RIPA) lysis buffer (P0013B; Beyotime, China) with protease inhibitor cocktail. An equal amount of protein (50 μg) was separated by 10% sodium dodecyl sulfate polyacrylamide gel electrophoresis (SDS-PAGE) and then transferred onto polyvinylidene difluoride (PVDF; Bio-Rad, USA) membranes. After the membranes were blocked with 5% nonfat milk powder, they were incubated overnight at 4 °C with the following primary antibodies: mouse anti-Bax (1:1 000, sc-7480; Santa Cruz, USA), mouse anti-Bcl-2 (1:1 000, sc-7382; Santa Cruz, USA), mouse anti-PCNA (1:1 000, BM0104; Bosterbio, China), or rabbit anti-GAPDH (1:2 000, 10494–1-AP; Proteintech, China). The corresponding horseradish peroxidase-conjugated secondary antibodies (1:5 000, SA00001–1, SA00001–2; Proteintech, China) were added to the membranes for 2-h incubations. After three washes, the membranes were visualized by high-sig ECL western blotting substrate (Tanon, China). The experiments were repeated at least three times with similar results.

### TUNEL staining

To determine the development of follicles, TUNEL assays were carried out by a commercial kit (In Situ Cell Detection Kit-POD, Roche, Germany) according to the manufacturer’s instructions. Paraffin-embedded ovarian sections were deparaffinized and hydrated, and then they were processed for blocking endogenous peroxidase activity and antigen retrieval pretreatment. After the slides were blocked with 1% bovine serum albumin for 2 h, they were incubated with the TUNEL reaction mixture in a humid chamber for 1 h at 37 °C. Positive signals were developed with DAB solution (ZLI-9018; ZSGB-BIO, China). Counterstaining was conducted with hematoxylin. Finally, the slides were observed under a light microscope.

### Measurement of oxidative stress

To analyze oxidative stress markers, the ovaries were ultrasonically homogenized, and the supernatant was obtained by centrifugation at 10 000 g for 15 min. An ELISA kit (DYC3419–2, R&D Systems, Inc. USA.) was used for estimating the level of SOD2. Additionally, GSH-Px and MDA measurements were respectively performed by Glutathione Peroxidase (GSH-PX) assay kit (A005, Nanjing Jiancheng Bioengineering Institute, China) and Malondialdehyde (MDA) assay kit (A003, Nanjing Jiancheng Bioengineering Institute, China).

### Statistical analysis

All data were expressed as the mean ± standard error of the mean (SEM). SPSS statistical software (SPSS Inc., Chicago, IL, USA) was used for analysis. A value of *P* < 0.05 was considered to be statistically significant. The normal distribution of the quantitative variables was tested using the Shapiro-Wilk test. If univariate quantitative data of groups met the normal distribution and homogeneity of variance criterion, one-way analysis of variance (ANOVA) followed by Tukey post-hoc test or independent-samples test were used. If not, Kruskal-Wallis test was used. All experiments were repeated at least three times.

## Results

### Ovarian weight and estrus cycle

**Fig. 2:**
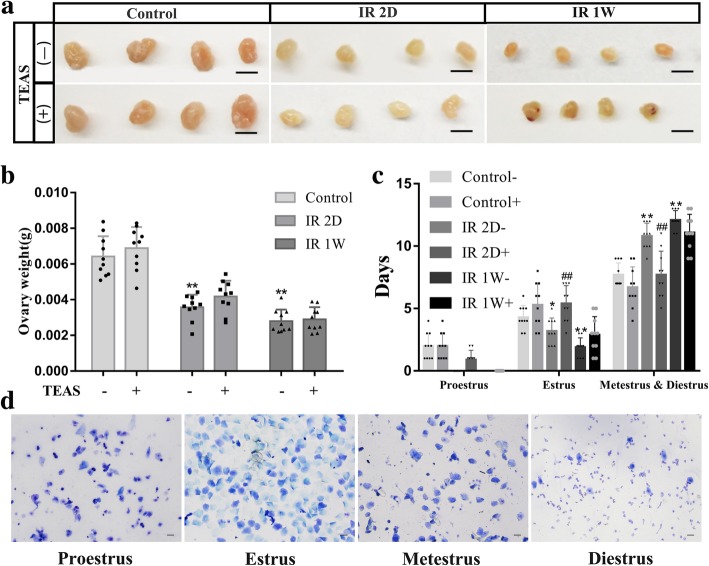
Comparison of ovarian function among groups after irradiation and/or TEAS. **a** The actual ovarian sizes in each group. **b** The changes in ovarian weight. **c** Effect of irradiation and TEAS treatment on estrous cycle. **d** Representative staining of vaginal smears was shown. Total *n* = 60. The data represent the mean ± SEM. *P < 0.05, ** *P* < 0.01 vs. control- group. ##*P* < 0.01 vs. IR 2D- group. The scale bars represent 2 mm (**a**), 80 μm (**d**)

As shown in Fig. 2a, the ovarian sizes of mice in the IR 2D and IR 1 W groups were smaller than those in the control group. Similarly, compared with the control- group, the ovarian weight significantly decreased in the IR 2D (*P* < 0.001) and IR 1 W (*P* < 0.001) groups. However, no difference in the ovarian weight was observed between the IR 2D- and IR 2D+ groups (*P* = 0.103) or between the IR 1 W- and IR 1 W+ groups (*P* = 0.227) (Fig. 2b).

Estrus cycles were determined via vaginal smear analysis during 2 weeks of TEAS treatment. As shown in Fig. 2c, irradiation induced irregular estrous cycle patterns, in which majority of the irradiation with mock TEAS treatment mice (IR 2D- and IR 1 W- groups) were in persistent metestrus and diestrus phases (both *P* < 0.001), and in shorten estrus (both *P* < 0.05). Compared with the IR 2D- group, the estrus days of IR 2D+ group were longer (*P* < 0.001), and the metestrus and diestrus phases were shorter (*P* < 0.001) after TEAS treatment. Interestingly, the proestrus phase was not different significantly in two groups. However, there was no difference in the estrus cycle patterns between the IR 1 W- and IR 1 W+ groups.

### Measurement of serum AMH level

**Fig. 3:**
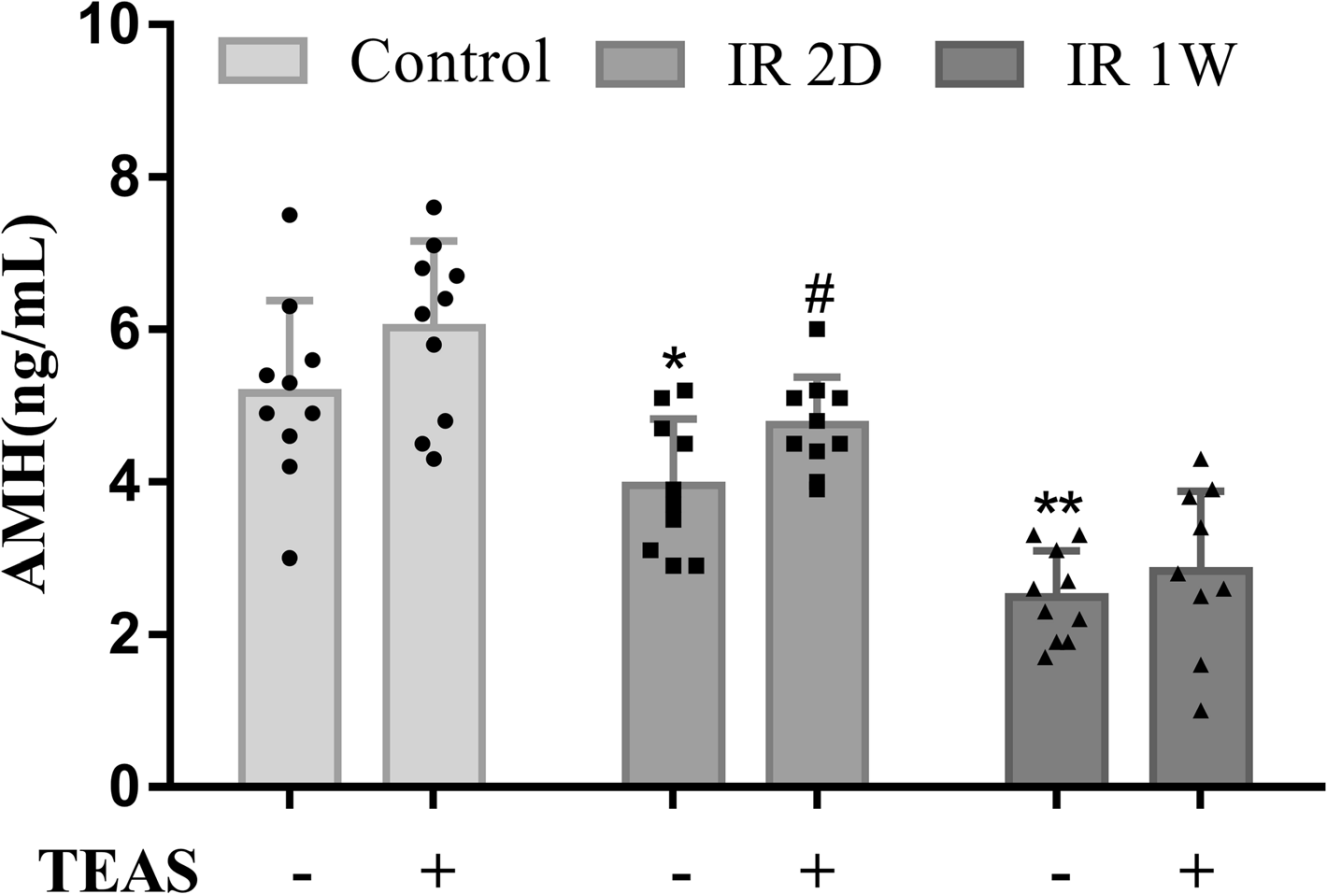
The effect of TEAS on irradiation-induced serum AMH level. The data represent the mean ± SEM. * *P* < 0.05, ** *P* < 0.01 vs. control- group; # *P* < 0.05 vs. IR 2D- group

As shown in Fig. 3, compared with the control- group, the serum AMH level was decreased in the IR 2D- (*P* = 0.018) and IR 1 W- (*P* < 0.01) groups. Moreover, the AMH concentration in the IR 2D+ group was obviously higher than that in the IR 2D- group (*P* = 0.031). However, no statistically significant difference was found between the IR 1 W- and IR 1 W+ groups (*P* = 0.372).

### Follicle counts and ovarian morphology

**Fig. 4:**
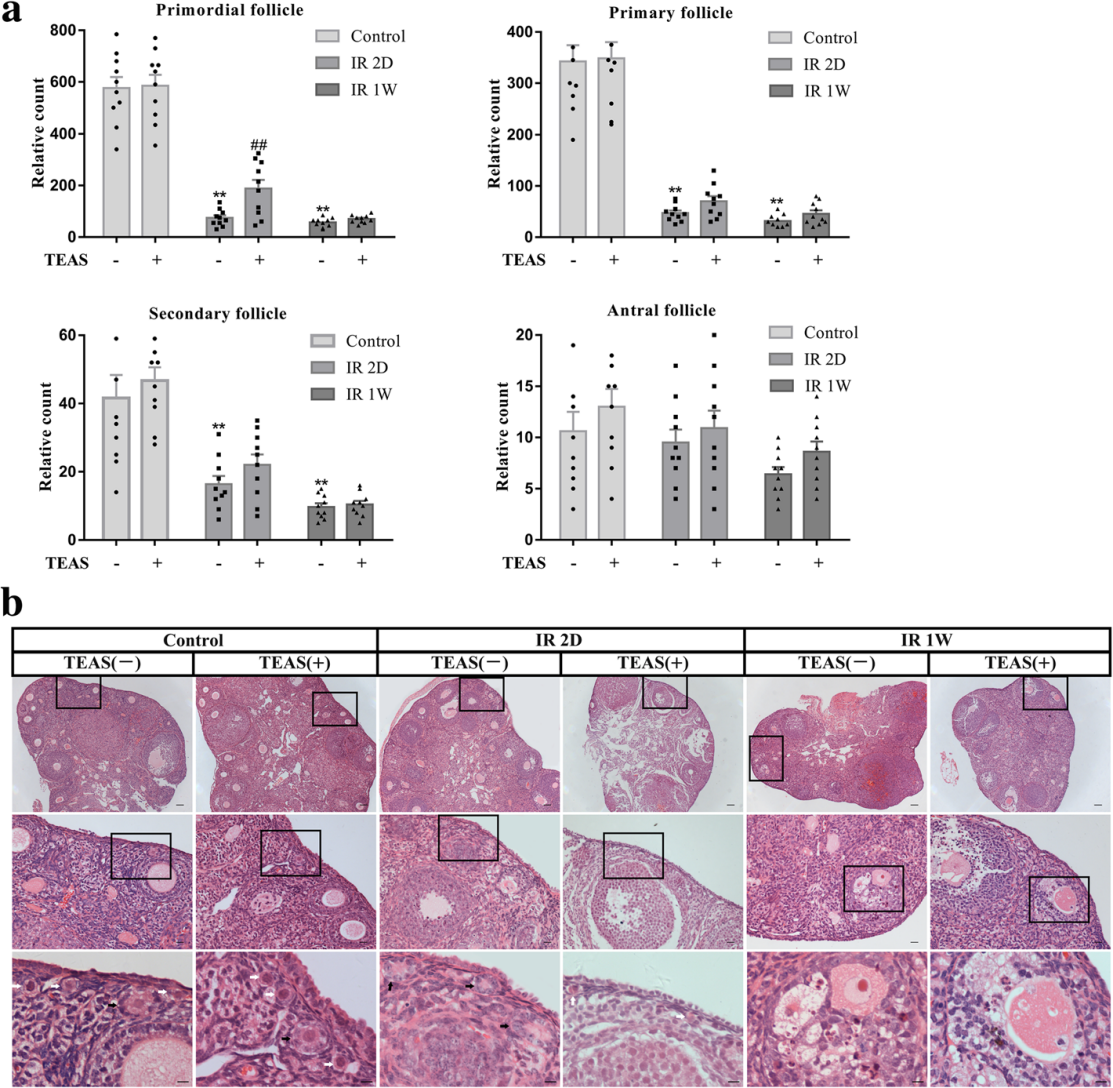
The effect of TEAS on irradiation-induced follicle count and ovarian morphology. **a** The numbers of primordial, primary, secondary, and antral follicles in each group from the ovary with H&E staining (*n* = 10/group). **b** Histological examinations of ovaries in each group were analyzed by H&E staining. White arrows: primordial follicles, black arrows: primary follicles. The data represent the mean ± SEM. ** *P* < 0.01 vs. control- group; ## *P* < 0.01 vs. IR 2D- group. A white arrow shows the primordial follicles, and a black arrow shows the primary follicles. The scale bars represent 80 μm (first line), 20 μm (second line), and 10 μm (third line)

As shown in Fig. 4a, compared with the control- group, significantly decreased numbers of primordial, primary and secondary follicles were seen in the IR 2D- and IR 1 W- groups (*P* < 0.01), and no difference was found in the number of antral follicles (IR 2D-: *P* = 0.637, IR 1 W-: *P* = 0.053).

For the primordial follicle count, a statistically significant difference was found only for the IR 2D+ group, which showed an increased level compared with that of the IR 2D- group (*P* = 0.004), and there was no difference between the IR 1 W- and IR 1 W+ groups (*P* = 0.104). Additionally, no effect on primary, secondary or antral follicle counts were observed between the IR 2D- and IR 2D+ or between the IR 1 W- and IR 1 W+ groups (*P* > 0.05).

As shown in Fig. 4b, the morphology of the ovaries was obviously damaged by irradiation. The control group showed normal ovarian histological structure of the cortex and medulla with multiple follicles of different stages, intact oocytes, and corpus luteum. In the IR 2D- group, the irradiated ovarian sections showed few follicles with hemorrhage in the cortex, ovarian interstitial hyperplasia, and thickening blood vessels in the ovarian cortex. Many small primary follicles are atretic with degenerating oocytes and granulosa cells (black arrows). However, the TEAS treatment improved histological appearance, which showed less interstitial fibrosis, well-developed corpus luteum and multiple primordial follicles with intact oocytes (white arrows) in IR 2D+ group.

### Expression of Bax, Bcl-2, PCNA by Western blotting

**Fig. 5:**
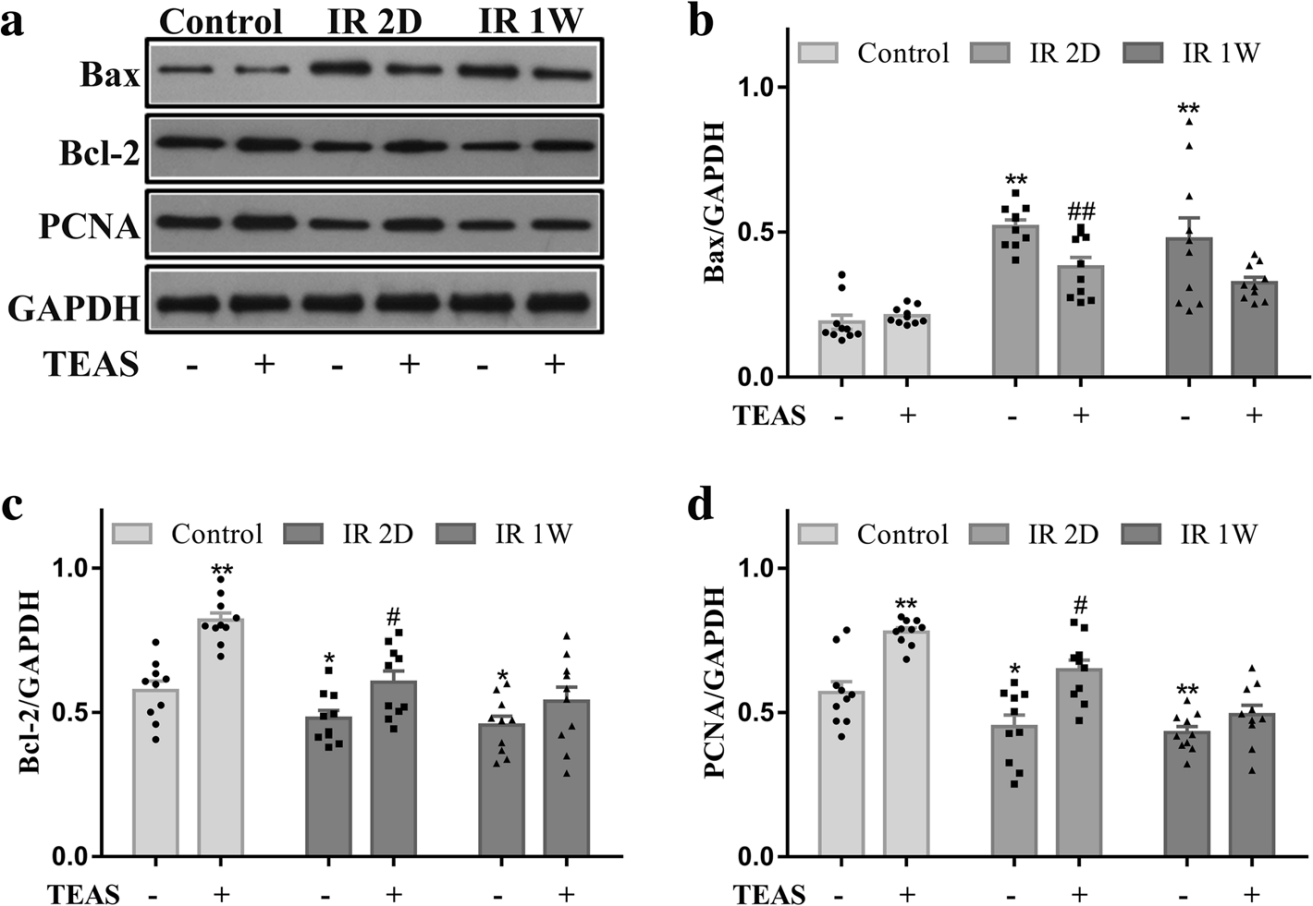
The effect of TEAS on irradiation-induced expression of Bax, Bcl-2 and PCNA proteins in ovary. **a** The protein expression levels of Bax, Bcl-2, PCNA were determined by western blot analysis. **b**–**d** Quantitative analysis of total proteins was represented using a bar graph. The data represent the mean ± SEM. * *P* < 0.05, ** *P* < 0.01 vs. control- group; # *P* < 0.05, ## *P* < 0.01 vs. IR 2D- group

Protein analysis of whole ovaries from 8-week-old mice after irradiation and/or TEAS treatment was conducted to assess changes in Bax, Bcl-2, and PCNA proteins. With GAPDH used as a loading control, western blot analysis showed that the relative level of Bax in IR 2D- and IR 1 W- groups were 3-fold higher than that in the control- group (IR 2D-: *P* < 0.001, IR 1 W-: *P* = 0.002), but the increase in the IR 2D- group was evidently reduced by the combination with TEAS in the IR 2D+ group (*P* = 0.003), and no difference was found between the IR 1 W- and IR 1 W+ groups (*P* = 0.062; Fig. 5b). Similarly, Bcl-2, which is known as an anti-apoptotic factor, showed significantly lower expression in the IR 2D- and IR 1 W- groups compared with the control- group (IR 2D-: *P* = 0.036, IR 1 W-: *P* = 0.015), but the expression of Bcl-2 in the IR 2D+ group was significantly increased in contrast to that in the IR 2D- group (*P* = 0.017; Fig. 5c). Additionally, the relative level of PCNA protein was markedly lower in the IR 2D- and IR 1 W- groups than that in the control- group (IR 2D-: *P* = 0.045, IR 1 W-: *P* = 0.005), and yet, in the IR 2D+ group, the level of PCNA protein was obviously higher than that in IR 2D- group (*P* = 0.017; Fig. 5d). No difference was found between the IR 1 W- group and the IR 1 W+ group (*P* = 0.134).

### TUNEL staining

**Fig. 6:**
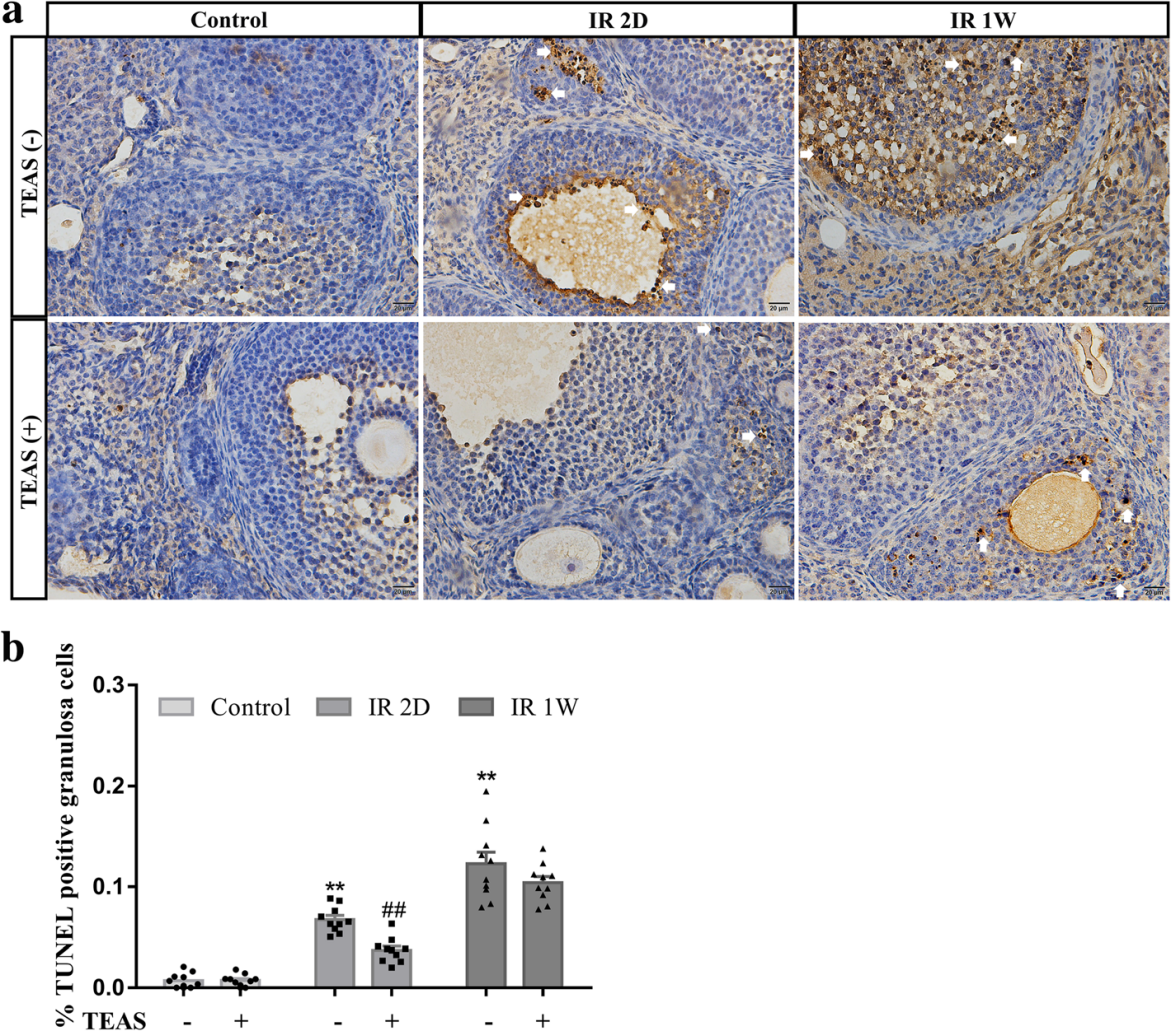
In situ end labeling of DNA fragmentation on ovary sections. **a** Representative image of TUNEL immunostaining in granulosa cells of follicles. **b** Graphs show the mean ± SEM percentage of follicles with TUNEL-positive granulosa cells. * *P* < 0.05, **P < 0.01, vs. control- group; # *P* < 0.05, ## *P* < 0.01, vs. IR 2D- group. *n* = 10 mice/group. Arrow: TUNEL-positive granulosa cells, scale bars: 20 μm

As shown in Fig. 6a, immunohistochemical detection of TUNEL was assessed to evaluate the ovarian granular cells apoptosis. The number of immunostained positive granular cells was quantified and the results are represented in Fig. 6b. In the ovaries of the control- group, the cells showed weakly positive TUNEL formation, and high TUNEL formation was observed in the IR 2D- and IR 1 W- groups (IR 2D-: 6.67 ± 0.42% vs. 0.72 ± 0.23%, *P* < 0.001; IR 1 W-: 12.28 ± 1.17% vs. 0.72 ± 0.23%, *P* < 0.001). However, the percentage of TUNEL-positive cells in the IR 2D+ mice was significantly lower than that in IR 2D- group (3.72 ± 0.39% vs. 6.67 ± 0.42%, *P* < 0.001). No difference was found between the IR 1 W- group and the IR 1 W+ group (12.28 ± 1.17% vs. 10.41 ± 0.59%, *P* = 0.171).

### Oxidative stress markers

**Fig. 7:**
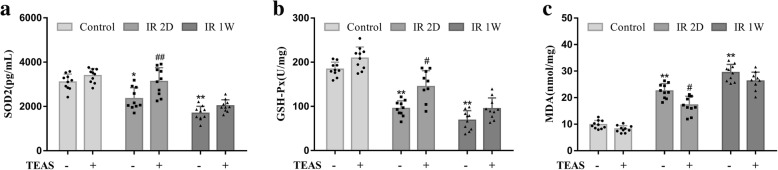
The effect of TEAS on oxidative stress in ovaries of mice subjected to whole-body irradiation. **a** SOD2 activities in wet ovaries were analyzed by ELISA. **b** GSH-Px activities in wet ovaries were represented as U/mg of protein. **c** MDA levels in wet ovaries were represented as nmol/mg of protein. The data represent the mean ± SEM. * *P* < 0.05, ** *P* < 0.01 vs. control- group; # *P* < 0.05, ## *P* < 0.01 vs. IR 2D- group

Irradiation-induced oxidative stress in mouse ovary was evaluated by assessing SOD2 and GSH-Px activities as well as MDA levels. As shown in Fig. 7, compared with control- group, the activities of SOD2 and GSH-Px obviously decreased whereas the MDA level increased significantly in both IR 2D- and IR 1 W- groups. However, the SOD2 and GSH-Px activities of IR 2D+ mice significantly increased than those in IR 2D- group. Moreover, the MDA level was significantly lower in IR 2D+ mice than that in IR 2D- group. There were no differences in SOD2, GSH-Px and MDA levels between IR 1 W- and IR 1 W+ groups.

## Discussion

Several studies have shown that traditional acupuncture/TEAS can alleviate radiotherapy-related side effects [23, 30]. However, its potential in vivo radioprotective effect on the ovarian function is still uncertain. In the present study, the molecular mechanisms underlying the potential ovarian radioprotective effect of TEAS has been assessed by studying its effects on AMH secretion, follicular counts and different markers of apoptosis, proliferation, oxidative stress and folliculogenesis in an experimental model of radiation-induced ovarian failure.

AMH is produced by the granulosa cells in late prenatal and small antral follicles, and circulating AMH reflects the number of primordial follicles [31]. AMH was also considered as a sensitive indicator of the iatrogenic ovarian failure in human and experimental studies [32, 33]. Alipour et al. indicated that AMH is a better marker of POF [34]. In our present study, irradiated mice showed lower levels of serum AMH than those of the control group. Cotreatment with TEAS markedly increased AMH levels in the 2 days post irradiation group. However, irradiation in the late TEAS group (IR 1 W+) did not alleviated the decrease in the AMH level induced by irradiation. Human and experimental studies have previously reported that, after exposure to irradiation, the low or undetectable level of AMH was checked [35]. These results are in accordance with an earlier study that showed that electroacupuncture pretreatment increased the AMH level in rats with POI induced by 4-vinylcyclohexene diepoxide [36].

In addition to AMH level assessment, the different stages of follicles were determined, and the morphological analyses of the ovaries were carried out. Our results found the exhaustion of the primordial follicle and the reduction of primary and secondary follicles after irradiation. These results agreed with the study that reported that exposure to radiotherapy causes increased recruitment of primordial follicles, finally leading to the excessive expenditure of the primordial follicle pool and to premature ovarian failure/insufficiency (POF/POI) [37, 38]. Moreover, the primordial follicle count in the group treated with TEAS 2-days post irradiation showed a significant increase compared with the untreated group. Zheng et al. reported that TEAS as an adjunct treatment was applied to ameliorate the level of gonadotropin and the ovarian microenvironment and to achieve more high-quality oocytes and embryos for patients with diminished ovarian reserve during assisted reproductive technology [39]. The administration of TEAS in polycystic ovary syndrome (PCOS) model rats also has beneficial effects on the function of the hypothalamus-pituitary-ovary axis [40]. However, mice in the 1 week post irradiation group only had a few follicles left in the mouse ovary, and TEAS had no significant effect on irradiation-induced follicular damage in the 1 week post irradiation group, probably because the post irradiation time was too long to destroy all of the follicles. It has been reported that irradiation with 50 cGy space radiation depleted 99% of ovarian follicles at 8 weeks after irradiation in adult C57BL6 mice [38]. Thus, the damage to follicles that was induced by irradiation was more severe with increasing post irradiation time, and the earlier the radiotherapy mice received TEAS treatment, the better.

It has been reported that irradiation induces follicular cell apoptosis and impairs ovarian functions [20]. The present study showed that the expression of PCNA in irradiated mouse ovary was markedly decreased followed by an increase of Bax proteins and a decrease of Bcl-2 proteins, and these defects were obviously improved in the group that received TEAS treatment 2 days after irradiation. In accordance with our results, studies have offered evidence of a dysregulation of the Bcl-2/Bax ratio including a study showing that irradiation increased Bax protein expression with a concurrent decrease in Bcl-2 protein expression in rats [41], and in the ovarian follicles of the irradiated rat the proliferation of granulosa cells was markedly reduced by PCNA staining [20]. TEAS significantly inhibited apoptosis and promoted proliferation induced by irradiation. Our results were in accordance with previous studies; TEAS can raise the Bcl-2/Bax ratio and reduce neuron apoptosis in the hippocampus of beagles undergoing general anesthesia or controlled hypotension surgery [42]. Studies have demonstrated that follicular atresia is caused by the apoptosis of granulosa cells, and irradiation induces cell apoptosis and impairs ovarian functions [7, 15]. Our study demonstrated that the frequency of TUNEL-positive cells in the granulosa of follicles was significantly lower than that in the granulosa of atretic follicles in whole-body radiation mice. However, cotreatment with TEAS at 2 days post irradiation (IR 2D+) markedly decreased the reactivity of TUNEL in the granulosa cells of the radiation group. It was reported that in rat ovaries, acupuncture can inhibit granulocyte apoptosis [43]. In this study, TUNEL analysis showed that TEAS reduced irradiation-induced granulosa cell apoptosis in mouse ovaries.

Ionizing radiation impairs the cellular function mainly by generating ROS [9], which can induce the release of cytochrome c and other apoptogenic factors from the mitochondria, and can eventually lead to cellular apoptosis [9]. Accumulating evidence show that excessive ROS generation triggers antral follicular atresia by causing granulosa cell apoptosis [10]. The balance between ROS and the anti-oxidants are required for ovarian function. In our study, ovarian SOD2 and GSH-Px were significantly decreased, and MDA was drastically elevated following a 4 Gy dose of X-irradiation. However, TEAS treatment initiated at 2 days after irradiation significantly increased the levels of SOD2 and GSH-Px and decreased the level of MDA, leading to an improved antioxidant capacity of ovarian tissue. Recent studies have reported 7that electroacupuncture pretreatment can attenuate ovarian oxidative stress in rodent models [44, 45]. Accordingly, our study proved that blocking oxidative stress is another mechanism by which TEAS rescues the X-irradiation-induced ovarian follicular loss.

## Conclusions

The present study demonstrates that TEAS treatment 2 days post irradiation significantly protects the primordial ovarian follicle counts and increases AMH secretion, while it inhibits apoptosis through decreasing the expression of Bax, increasing the expressions of Bcl-2, reducing granulosa cell apoptosis and inhibiting the formation of ROS. However, the present study had several limitations. Further research will continue to evaluate the mechanism of TEAS treatment in irradiation-induced model mice.

## Data Availability

All the data is contained in the manuscript.

## Abbreviations

AMH: Anti mullerian hormone
Bax: BCL2-Associated X
Bcl-2: B-cell lymphoma-2
DAB: Diamino benzidine
DNA: Deoxyribonucleic acid
ECL: Enhanced chemiluminescence
ELISA: Enzyme-linked immuno sorbent assay
ESR1: Estrogen receptor α gene
GSH-PX: Glutathione Peroxidase
H&E: Hematoxylin and eosin
HFM1: Helicase for meiosis 1
MDA: Malondialdehyde
PCNA: Proliferating cell nuclear antigen
PCOS: Polycystic ovary syndrome
POF: Premature ovarian failure
POI: Premature ovarian insufficiency
PVDF: Polyvinylidene difluoride
RIPA: Radio immunoprecipitation assay
ROS: Reactive oxygen species
SDS-PAGE: Rodium dodecyl sulfate polyacrylamide gel electrophoresis
SOD2: Superoxid dismutase 2
TEAS: Transcutaneous electrical acupoint stimulation
TUNEL: Terminal deoxynucleotidyl transferase dUTP nick end labeling
